# Surface Micromorphology of Experimental Composites Doped with Bioactive Glass After Different Storage Times

**DOI:** 10.3390/jfb16040140

**Published:** 2025-04-14

**Authors:** Leonardo Svellenti, Moritz Tanner, Andrea Gubler, Matej Par, Thomas Attin, Phoebe Burrer, Tobias T. Tauböck

**Affiliations:** 1 Department of Periodontology, Endodontology, and Cariology, University Center for Dental Medicine Basel UZB, University of Basel, Mattenstrasse 40, 4058 Basel, Switzerland; 2 Clinic of Orthodontics and Pediatric Dentistry, Center for Dental Medicine, University of Zurich, Plattenstrasse 11, 8032 Zurich, Switzerland; moritz.tanner@zzm.uzh.ch; 3 Clinic of Conservative and Preventive Dentistry, Center for Dental Medicine, University of Zurich, Plattenstrasse 11, 8032 Zurich, Switzerland; andrea.gubler@zzm.uzh.ch (A.G.); thomas.attin@zzm.uzh.ch (T.A.); phoebe.burrer@zzm.uzh.ch (P.B.); tobias.tauboeck@zzm.uzh.ch (T.T.T.); 4 Department of Endodontics and Restorative Dentistry, University of Zagreb School of Dental Medicine, Gunduliceva 5, 1000 Zagreb, Croatia

**Keywords:** dental materials, resin composite, bioactive glass fillers, surface micromorphology, SBF storage, ultrasonic cleaning

## Abstract

Objective: To evaluate the surface micromorphology of bioactive glass-modified resin composite materials after storage in simulated body fluid for different periods of time and ultrasonic cleaning. Materials and methods: A resin composite material (Heliomolar Flow, Ivoclar Vivadent) was modified by incorporating 10 or 20 wt% of bioactive glass 45S5. The unmodified conventional composite (0 wt% bioactive glass) served as the control. Surface morphology of light-cured composite specimens was examined by profilometry both before and after storage in simulated body fluid (SBF; pH = 7.4, t = 37 °C) for 0, 3, 7, or 30 days, and surface roughness (Ra) was recorded. After storage, ultrasonic cleaning (UC) of the specimens was performed for 10 min in an ultrasonic bath filled with deionized water, and the profilometric measurements were subsequently repeated. In addition, the surfaces of specimens were examined by scanning electron microscopy (SEM). Results: Directly after specimen preparation, the Ra values of the composites modified with bioactive glass were similar to those of the conventional composite (0 wt% bioactive glass). A longer immersion in SBF and higher added concentrations of bioactive glass led to an increase in surface roughness. SEM examination revealed that precipitates were formed on the surfaces of specimens containing bioactive glass after exposure to SBF for at least 7 days. The density of these precipitates increased with exposure time and added bioactive glass content. After subsequent ultrasonic cleaning, a significant Ra reduction was observed for specimens containing 10 and 20 wt% bioactive glass and stored for 30 days (*p* < 0.001). For the resin composite material doped with 20 wt% bioactive glass particles, UC revealed a significant Ra reduction at all time points. Conclusion: The increase in the surface roughness of bioactive glass-modified composites after storage in SBF might be partly attributed to precipitate formation on their surfaces. After ultrasonic cleaning, surface roughness was still increased, indicating poorer surface quality compared to conventional composite.

## 1. Introduction

Composite materials are considered the material of choice for direct dental restorations. Besides their numerous advantages, such as defect-oriented restorative approaches and adhesive luting, composite materials still exhibit polymerization shrinkage [1]. Although advances in material technology have reduced this effect over the past decades [2], marginal gap formation at the tooth-restoration interface remains a concern and may contribute to the development of secondary caries, one of the most common reasons for the replacement of restorations [3].

During the second half of the last century, the discovery of bioactive glasses by L.L. Hench paved the way for modern, biomaterial-driven regenerative medicine [4]. The synthetically manufactured “Bioglass 45S5” was the first product to be shown to form a bond with living bone and, furthermore, to actively stimulate osteogenesis [5,6]. Bioactive glasses consist of different silicate-based compositions, and their mechanism of action is mainly explained by the release of several ions, such as calcium and phosphate [5,7]. When exposed to biological fluids, bioactive glasses form a hydroxyapatite-like surface layer and interact with surrounding tissues [6,8]. Today, these glasses are approved and routinely used for a wide range of medical applications, including dentistry [9,10].

Taking into account the fact that dental hard tissues largely consist of hydroxyapatite and that their structural integrity is subject to alternating and continuous de- and remineralization processes, the idea of integrating calcium- and phosphate-releasing compounds such as bioactive glasses into dental restorative materials seems obvious [11]. Incorporating bioactive glass into a BisGMA/TEGDMA resin system affects various physicochemical properties. For example, viscosity increases with particle loading but remains below that of conventional flowable composites. Additionally, water sorption and pH changes are influenced, while microhardness and degree of conversion show only minor variations [11]. The addition of bioactive glass particles to composite materials could, therefore, offer various advantages, and their remineralizing and antibacterial effects have been shown in several studies [12,13]. Yli-Urpo et al. [12] described not only the formation of calcium-phosphate precipitates on the surface of glass ionomer cement containing bioactive glass but also detected mineral deposits in the dentinal tubules of the adjacent tooth structure, suggesting a remineralizing effect on the tooth surface. Furthermore, Jang et al. [14] showed that a composite containing bioactive glass significantly increased the microhardness of the adjacent demineralized dentin. Recent studies on bioactive glass-containing fissure sealants have demonstrated their ability to neutralize acidic solutions and have revealed appropriate mechanical and physical properties [15].

Modifying resin composite materials with bioactive glass could, therefore, be a promising approach to prevent the formation of secondary caries [13,14]. However, the question remains whether their physicomechanical properties can keep up with those of conventional composite materials, as ion-releasing fillers are known to gradually degrade when exposed to an aqueous environment [16].

For long-lasting dental restorations, it is important that the surface structure remains intact and smooth over time to decrease the risk of biofilm adhesion. Since bioactive glasses release or form various substances on the surface [11,17], it is particularly important to evaluate whether the surface micromorphology and roughness of composites modified with bioactive glass are comparable to that of conventional composites.

Therefore, the aim of the present study was to assess the surface micromorphology and roughness of experimental composites modified with different amounts of bioactive glass 45S5 in relation to conventional composite after different storage periods in SBF. In addition, the influence of subsequent ultrasonic cleaning after each period of SBF storage was investigated. In the null hypothesis, it is assumed that the ion-releasing composite materials provide similar surface micromorphology and roughness as conventional unmodified composites after storage and ultrasonic cleaning.

## 2. Materials and Methods

### 2.1. Specimen Preparation

**Figure 1:**
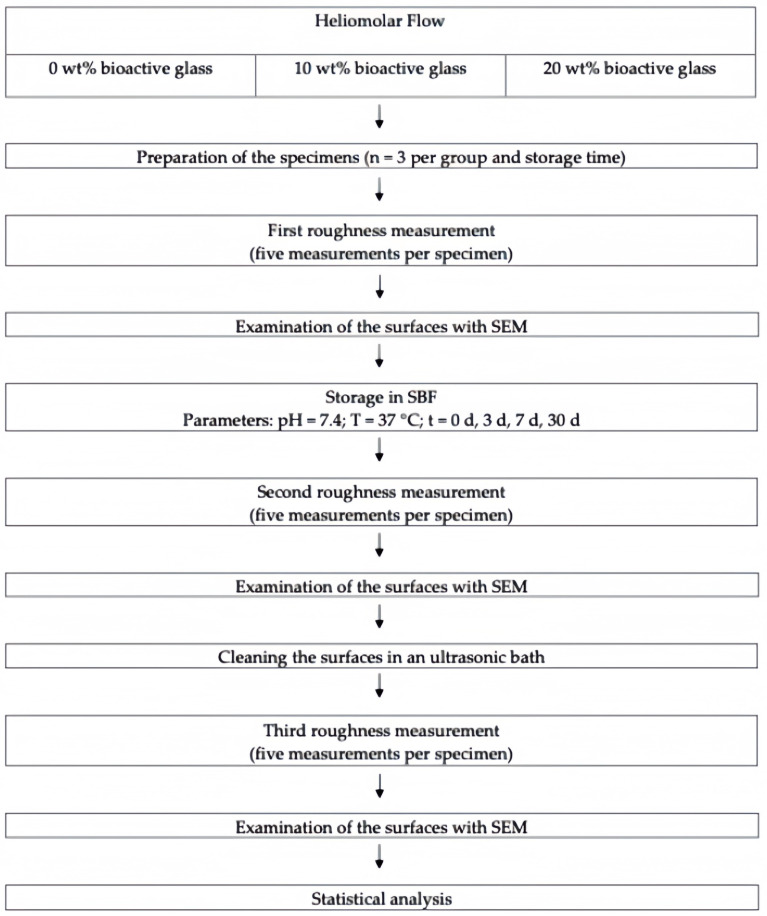
Study design diagram.

As an additive, bioactive glass 45S5 (Schott, Mainz, Germany) was used, which is classified as a micro-sized filler with an average particle size of 5.6 μm. As a composite material, Heliomolar Flow (Ivoclar Vivadent, Schaan, Liechtenstein; shade A2) was used. The manufacturers’ information about the materials is given in Table 1. The study design is depicted in Figure 1.

Bioactive glass was incorporated into the composite material using a high-speed centrifuge (Speedmixer DAC 150 FV2; Hauschild Engineering, Hamm, Germany).

First, the amount of bioactive glass needed, which varied according to the groups between 10 and 20 wt%, was placed in a light-tight container by using a standard precision scale (Mettler Toledo, Greifensee, Switzerland). Subsequently, it was filled with the composite material for a total mass of 2 g. The containers were mixed in the centrifuge for 2 min. The composite material was then stored in the dark until further processing within the next 6 h.

For specimen preparation, the prepared composite material with or without added bioactive glass was placed in custom-made round polystyrene molds, each with a diameter of 6 mm and a height of 2 mm. A glass plate (Thermo Scientific Menzel X50, Gerhard Menzel, Braunschweig, Germany) was placed on the lower surface of the molds, and the upper surface was covered by a transparent plastic matrix (Hawe Stopstrip, Kerr Dental, CA, USA), and another glass plate so that the composite material was pressed in between. Light-curing was performed for 40 s using the Bluephase G2 curing unit (Ivoclar Vivadent, Schaan, Liechtenstein), which was placed in direct contact with the 1 mm thick glass plate covering the upper surface of the molds. The applied irradiance (1170 mW/cm^2^) was checked repeatedly with a calibrated power meter (FieldMaxII-TO, Coherent, Santa Clara, CA, USA).

After light-curing, the upper surfaces of the specimens were ground under continuous water cooling with 2000 grit SiC paper and 4000 grit SiC paper (Struers, Copenhagen, Denmark) at a pressure of 2 N for 15 s. To remove residual grinding debris and ensure a clean surface for subsequent analysis, specimens were first briefly swirled in ethanol and then in deionized water.

### 2.2. Storage in Simulated Body Fluid (SBF)

Specimens were then placed in SBF, which was prepared according to the recipe of Kokubo et al. [18] with an initial pH of 7.4. For the time points of 3, 7, and 30 days, the separate groups of specimens were stored in an incubator at 37 °C. SBF solution was changed three times per week. After reaching the respective time point, the specimens were stored in a vacuum for 24 h and then kept dry at room temperature until surface roughness measurements. For each group, three specimens were prepared. Sample size estimation was conducted using data from a preliminary study. Given the large effect size observed, a sample size of n = 3 per experimental group was determined to be sufficient to detect statistically significant differences among the materials with varying amounts of bioactive glass, with a statistical power over 95% at a significance level of 0.05. Statistical power analysis was performed using G*Power software (Version 3.1.9.7, Heinrich Heine University, Düsseldorf, Germany).

### 2.3. Evaluation of Surface Roughness

Before measuring surface roughness, each specimen was briefly swirled in deionized water and clamped in a specially designed carrier, which was brought into a defined starting position. The measurement was then carried out using a stylus perthometer (Form Talysurf-50, Taylor-Hobson, Leicester, UK). Five measurements per specimen were taken with a length of 2 mm and at a distance of 0.5 mm from each other. The recorded Ra value served as a basic metric in the evaluation of surface quality. It was calculated as the arithmetic mean of the absolute deviations between the roughness profile and the center line and is defined by the formula (Ra = 1/L ∫) |*z* (*x*)|*dx* [19].

### 2.4. Ultrasonic Cleaning Procedure

Because it was expected that calcium-phosphate precipitates would accumulate on the bioactive glass-modified composite specimens during storage in SBF, and to be able to examine the surfaces without precipitates and simulate a cleaning routine, an ultrasonic cleaning procedure was performed. For this purpose, the specimens were cleaned for 10 min in an ultrasonic bath (SW3H; Sonoswiss, Ramsen, Switzerland) in deionized water.

### 2.5. Scanning Electron Microscopy Analysis

To visualize the microstructure of the specimen surfaces and to evaluate the presence of precipitates, specimens were examined with a scanning electron microscope (SEM) before and after ultrasonic cleaning. Before the images were taken, specimens were dried in a vacuum for one day and then coated with carbon to increase conductivity. Scanning electron micrographs with a magnification of 5000× at 3 kV were taken using the Nova NanoSEM 450 (FEI Company, Hillsboro, OR, USA).

### 2.6. Statistical Analysis

Mean Ra values were calculated, and statistical analysis was performed using the SPSS statistical program (version 20, IBM, Armonk, NY, USA). The normality of the data was assessed using the Shapiro–Wilk test and normal Q–Q diagrams. As the inspection of the data in the Q–Q diagrams indicated departures from the assumption of normality, which were formally confirmed by statistically significant *p*-values of the Shapiro–Wilk test (*p* < 0.05), nonparametric methods were chosen. Specifically, a Wilcoxon signed-rank test for dependent specimens was used to compare Ra values before and after ultrasonic treatment within the same group of materials and storage period, while the Kruskal–Wallis test was employed for the analysis of Ra values between material groups with different amounts of bioactive glass within the same storage period. Additionally, the required sample size was estimated based on a priori power analysis to ensure adequate power for detecting significant differences, with the significance level set at 0.05. Due to the pronounced differences between the experimental materials, the effect size was determined to be large, with a Cohen’s d value of approximately 6.3. Based on this effect size and at a significance level of 0.05 for a two-tailed non-parametric test, the statistical power (the probability of detecting a statistically significant effect if one exists) was estimated to exceed 95% for three specimens per group. The high statistical power, which enabled the detection of significant differences despite the small sample size (n = 3), was primarily attributable to the large magnitude of the observed effect.

## 3. Results

### 3.1. Surface Roughness

**Figure 2:**
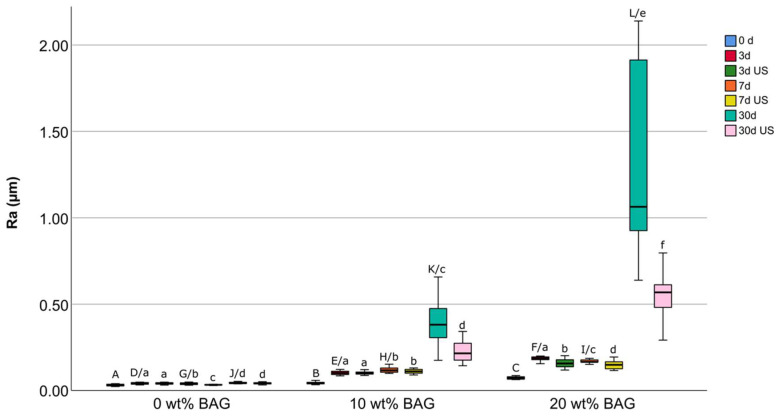

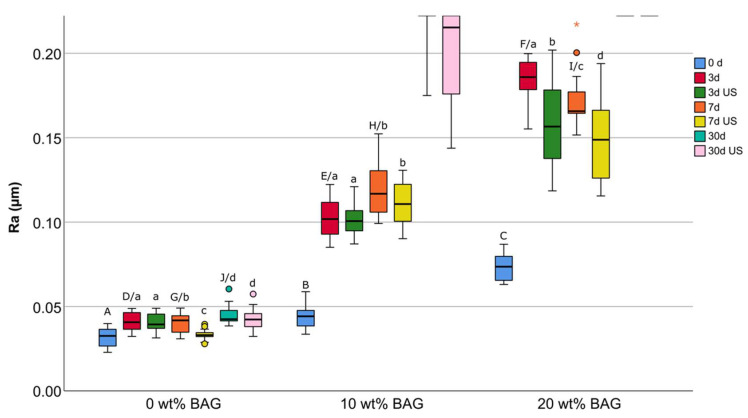
**above:** Boxplot with median Ra values, interquartile range, and upper/lower whisker of resin composite with 0 wt%, 10 wt%, or 20 wt% bioactive glass (BAG). The duration given in days corresponds to the storage time in simulated body fluid (SBF). US: Cleaning of the specimens in an ultrasonic bath. Groups marked with the same lowercase letters are not significantly different before and after US cleaning (within the same material group and the same storage time; *p* > 0.05). Groups with different BAG contents marked with the same capital letters are not significantly different from each other (within the same storage period; *p* > 0.05). **below:** Identical graphic with expanded scale to visualize lower Ra values. Outliers and extreme outliers are represented by circles and asterisks, respectively.

The Ra values at the different storage times (with and without ultrasonic cleaning) are shown in Figure 2.

For composite specimens without added bioactive glass (0 wt%), similar median Ra values were found over the different storage times, ranging from 0.0326 μm (interquartile range: 0.0130 μm) to 0.0425 μm (interquartile range: 0.0074 μm). For specimens with 10 wt% added bioactive glass, an increase in median roughness from 0.0442 μm (interquartile range: 0.0096 μm) to 0.3811 μm (interquartile range: 0.1907 μm) was observed after 30 days of storage in SBF. For specimens doped with 20 wt% bioactive glass, median Ra values also increased with storage time, ranging from 0.0736 μm (interquartile range: 0.0145 μm) at baseline to 1.0635 μm (interquartile range: 1.0296 μm) after 30 days of storage.

For all storage times (0, 3, 7, and 30 days), specimens without added bioactive glass showed significantly lower Ra values compared to specimens with 10 or 20 wt% added bioactive glass. Furthermore, specimens with 10 wt% bioactive glass showed significantly lower Ra values compared to those with 20 wt% bioactive glass, irrespective of storage time.

### 3.2. Surface Roughness After Ultrasonic Cleaning

In the group of composite materials without added bioactive glass, ultrasonic cleaning did not cause a significant decrease in Ra values, except after 7 days of storage. For specimens doped with 10 wt% bioactive glass, a significant decrease in median Ra values from 0.3811 μm (interquartile range: 0.1907 μm) to 0.2153 μm (interquartile range: 0.1088 μm) was observed after 30 days of storage followed by ultrasonic cleaning (*p* < 0.001). No significant Ra changes before and after ultrasonic cleaning could be observed at the other storage times. For specimens with 20 wt% added bioactive glass, a significant decrease in Ra values after ultrasonic cleaning was observed at each time point. In particular, after 30 days of storage, median Ra values decreased from 1.0635 μm (interquartile range: 1.0296 μm) to 0.5682 μm (interquartile range: 0.1360 μm) after ultrasonic cleaning (*p* < 0.001).

### 3.3. Scanning Electron Micrographs

**Figure 3:**
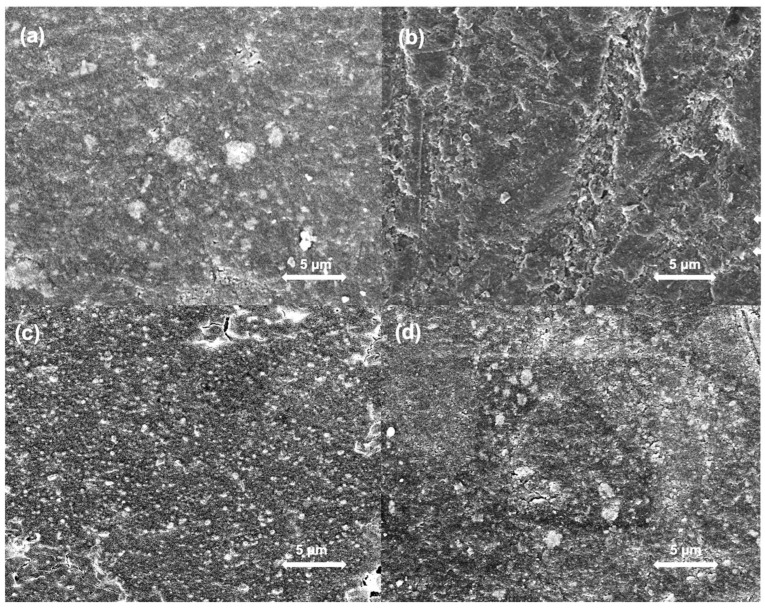
SEM images of specimens without added bioactive glass. (**a**) Specimens after 0 days of storage in SBF; (**b**) Specimens after 7 days of storage in SBF; (**c**) Specimens after 30 days of storage in SBF; (**d**) Specimens after 30 days of storage in SBF and subsequent ultrasonic cleaning.

**Figure 4:**
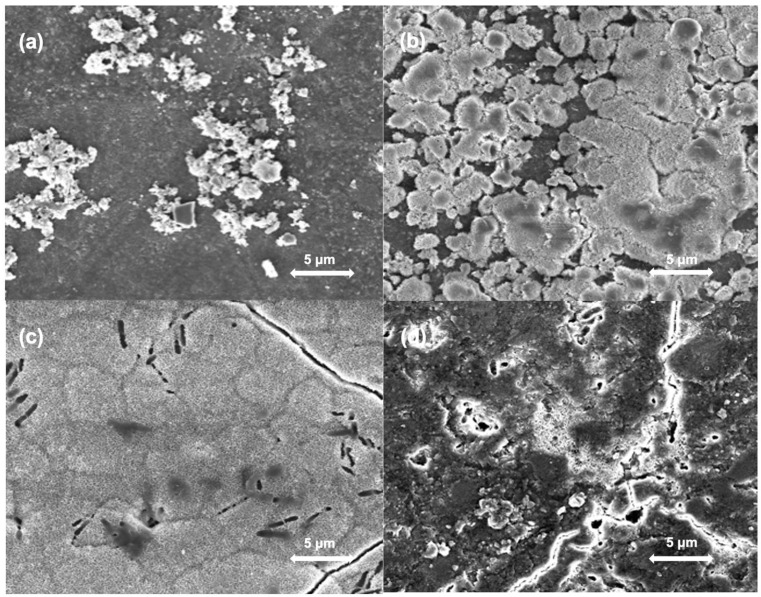
SEM images of specimens with 20 wt% bioactive glass. (**a**) Specimens after 0 days of storage in SBF; (**b**) Specimens after 7 days of storage in SBF; (**c**) Specimens after 30 days of storage in SBF; (**d**) Specimens after 30 days of storage in SBF and subsequent ultrasonic cleaning.

Figure 3 and Figure 4 provide representative SEM images of composites with 0 or 20 wt% bioactive glasses at different time points to illustrate the morphological differences observed in this study. When examining SEM images of specimens without added bioactive glass (Figure 3) taken after 7 days and 30 days of SBF storage without and with subsequent ultrasonic cleaning, surfaces showed only minor changes compared to their original state after preparation. The surfaces of specimens doped with 20 wt% bioactive glass showed a substantial change in their surfaces after an SBF storage period of 7 and 30 days (Figure 4). Increased precipitate formation could be observed on the surfaces. After 30 days, specimens have undergone the greatest change, with their surface being completely covered with precipitates. After ultrasonic cleaning, no more precipitates, yet a few smaller cracks were observed on the surface (Figure 4d).

## 4. Discussion

The present study examined the surface microstructure of composites doped with different amounts of micro-sized bioactive glass 45S5 at different time points and compared it with that of an unmodified composite. It was shown that the surface micromorphology of bioactive glass-modified composites immediately after specimen preparation and light-curing did not differ from that of the conventional composite. Furthermore, it was observed that with increasing content of bioactive glass and storage time in SBF, surface roughness increased. Finally, it was shown that although ultrasonic cleaning removed precipitates formed on composites containing bioactive glass, the roughness values were nevertheless higher compared to the initial state. The null hypothesis was rejected.

Prolonged storage in SBF resulted in an increase in surface roughness. This increase was more pronounced for specimens with higher concentrations of bioactive glass, which could be attributed to a more pronounced precipitation of calcium phosphates. The SEM micromorphological analysis additionally provided visual evidence for the formation of precipitates on the surfaces of bioactive glass-containing specimens after exposure to SBF. The precise composition of these precipitates was not analyzed in this study, but previous literature suggests they are likely calcium phosphate deposits. More precisely, several previous studies have shown that composites functionalized with bioactive glasses or other calcium- and phosphate-releasing components can induce the formation of calcium phosphate layers on their surface after storage in aqueous solutions [11,17]. The formation of hydroxyapatite-like precipitates on the surface could help seal marginal gaps [20] and promote the remineralization of tooth structures [21]. This ion-releasing behavior is a desirable property for dental restorative materials, as it can enhance the continuity of the interface between the restoration and the surrounding tooth structure [22]. The remineralizing and antimicrobial effects of bioactive glass-containing composites have been investigated and described in more detail in previous studies [12,14,17,20].

Subsequent ultrasonic cleaning of the specimens led in part to a reduction in surface roughness, which indicates a certain improvement in surface quality. Nevertheless, the roughness values after ultrasonic cleaning remained elevated compared to the initial values, indicating that either not all precipitates could be removed and/or that the surface had otherwise deteriorated, e.g., due to the dissolution of bioactive glass particles. Based on the SEM images showing cracks on the surfaces, the second hypothesis seems more likely. Nevertheless, it is important to note that these cracks might also be, at least in part, artifacts caused by the drying process. Composites containing bioactive glasses have been shown to absorb considerable amounts of water, resulting in swelling [23]. The subsequent drying step that had to be performed for surface roughness measurements rapidly removes this absorbed water, which might have resulted in cracking. While these cracks might contribute to the measured Ra values, they are also indicative of experimental artifacts, as such extreme drying conditions do not occur in vivo. This complicates the distinction between true increases in roughness and those induced by sample preparation. It should also be noted that the use of ion-releasing particles may represent a compromise. While these particles provide beneficial effects such as ion release, they can also increase surface roughness, which might facilitate bacterial adhesion [24]. A study on Giomers showed that the dissolution of reactive particles led to an increase in surface roughness, which correlated with greater bacterial adhesion [25]. This highlights the need to balance the beneficial effects of ion-releasing particles with their impact on surface properties.

For these reasons, further research is certainly needed to interpret the surface roughness measured in the present study in more detail. The median Ra values of composites modified with 10 wt% bioactive glass significantly decreased from 0.3811 μm (interquartile range: 0.1907 μm) to 0.2153 μm (interquartile range: 0.1088 μm) and those of composites modified with 20 wt% bioactive glass significantly decreased from 1.0635 μm (interquartile range: 1.0296 μm) to 0.5682 μm (interquartile range: 0.1360 μm) after 30 days of storage and after ultrasonic cleaning, respectively. For Ra values above 0.2 μm, it was shown that stronger biofilm formation occurs [26]. Considering this threshold value of 0.2 μm, composite specimens with 10 wt% bioactive glass exhibited a quite smooth surface after ultrasonic cleaning and almost reached this threshold. Specimens with 20 wt% bioactive glass did not show this effect, suggesting that the maximum amount of micro-sized bioactive glass used in this specific composite material should not exceed 10 wt%, or maybe even less, regarding surface micromorphology. In this context, the use of nano-sized bioactive glass particles instead of micro-sized particles could offer two additional advantages. Firstly, a smaller amount of nano-sized bioactive glass particles would be required to achieve the same ion-releasing effect, as their larger surface area enables a higher ion-release rate [27]. Secondly, nano-sized particles would likely have a smaller impact on surface roughness, as the pits formed during their dissolution are smaller compared to those caused by micro-sized particles [27].

An alternative to ultrasonic cleaning to maintain the surface of composite restorations doped with bioactive particles and still benefit from their effects could be frequent polishing. Furthermore, simple daily toothbrushing could potentially remove loosely bound plaque and improve surface smoothness. This approach would be more desirable because it relies on routine procedures performed by the patient rather than costly treatments performed by the dentist. Another factor to consider is the impact of surface treatments on the long-term roughness and stability of bioactive glass-modified composites. Investigating whether additional polishing protocols or protective coatings could mitigate the roughness increase after storage and cleaning would be beneficial.

A further notable point is that the behavior of the composites with bioactive glass was only tested in a neutral medium in this study. In more acidic media, surface degradation could be accelerated or more extensive [28]. Previous studies have shown that conventional composites immersed in acidic environments, such as soft drinks, experience accelerated degradation [29]. This effect may be amplified in composites doped with bioactive glass particles, given the faster dissolution of its fillers under acidic conditions. Additionally, a reduction in pH within plaque-retentive areas could be sufficient to further deteriorate the composite surface. Further investigation is necessary to comprehensively assess the long-term performance of composites containing bioactive glass in diverse pH environments, particularly under acidic conditions. Furthermore, the observation period of 30 days provides only an initial insight into material degradation. Longer-term studies are necessary to evaluate the durability of these composites under clinically relevant conditions, including prolonged exposure to oral fluids, pH fluctuations, and mechanical wear. This is particularly important, as environmental factors in the oral cavity may accelerate degradation processes and influence surface stability over time. However, as this study was conducted under in vitro conditions, its findings may not fully replicate the clinical setting. In vivo studies and clinical trials would be necessary to confirm the practical effectiveness and longevity of bioactive glass-modified composites. Moreover, the potential impact of bioactive glass on color stability, which is an important factor for aesthetic longevity, was not investigated and should be considered in future research.

A further limitation of the present study is the relatively small sample size. While the sample size was determined based on statistical power analysis, a larger number of specimens could provide a more comprehensive assessment of surface roughness variability. Future studies should consider increasing the sample size to enhance the generalizability of the findings. Additionally, our study was limited to the use of only one conventional composite material, which means that the results may not be directly transferable to other resin-based materials incorporating bioactive glass. Moreover, variations in bioactive glass composition and particle size may also influence surface roughness and remineralization behavior differently, highlighting the need for further investigations into alternative formulations that optimize their effects while minimizing surface degradation.

Furthermore, it should be noted that Ra values from other laboratory studies are only comparable to a limited extent since specimens were not manufactured and polished in the same way. Different storage methods and different media for the specimens are also conceivable. However, in previous studies on this topic, mainly SBF and phosphate-buffered saline (PBS) were used [11,14]. In a study by Jang et al. [14], no difference was found between storing the ion-releasing composite specimens in SBF or in PBS. Nevertheless, as the two media have different properties, it is also conceivable that they react differently with ion-releasing composite specimens depending on the study setup and material, so the results obtained in different solutions for different bioactive glasses cannot be directly compared [30].

## 5. Conclusions

Bioactive glass-modified experimental composites showed higher values of surface roughness after storage in SBF than the same composite without bioactive glass. Surface roughness increased with higher content of bioactive glass and longer storage times, most likely due to the precipitation of precipitates on the composite surface. After ultrasonic cleaning, surface roughness was still increased, indicating poorer surface quality compared to conventional composite. Further studies are needed to investigate which content of added bioactive glass provides an optimal balance between calcium phosphate release and stable surface properties.

## Figures and Tables

**Table 1:** Manufacturers’ information on the main materials used in the study.

Material	Composition	LOT No.	Manufacturer
Heliomolar Flow	Bisphenol-A-glycidyl-dimethacrylate, urethane dimethacrylate, triethylene glycol dimethacrylate, highly dispersed silicon dioxide, prepolymer, ytterbium trifluoride, stabilizers, catalysts and pigments	YZ1246	Ivoclar Vivadent, Schaan, Liechtenstein
Bioactive glass 45S5	45 wt% SiO_2_, 24.5 wt% CaO, 24.5 wt% Na_2_O, 6 wt% P_2_O_5_	SM528	Schott, Mainz, Germany

## Data Availability

The raw data supporting the conclusions of this article will be made available by the corresponding authors on request.
